# Obesity May Be Protective against Severe Perineal Lacerations

**DOI:** 10.1155/2016/9376592

**Published:** 2016-05-05

**Authors:** Diana Garretto, Brian B. Lin, Helen L. Syn, Nancy Judge, Karen Beckerman, Fouad Atallah, Arnold Friedman, Michael Brodman, Peter S. Bernstein

**Affiliations:** ^1^Department of Obstetrics & Gynecology and Reproductive Medicine, Stony Brook Medicine, Stony Brook University, Stony Brook, NY 11794, USA; ^2^Hospitals Insurance Company, Inc., New York, NY 10016, USA; ^3^Department of Obstetrics & Gynecology and Women's Health, Albert Einstein College of Medicine/Montefiore Medical Center, Bronx, NY 10461, USA; ^4^Bronx Lebanon Hospital, Bronx, NY 10457, USA; ^5^Maimonides Medical Center, Brooklyn, NY 11219, USA; ^6^Mount Sinai Beth Israel Hospital, New York, NY 10011, USA; ^7^Mount Sinai Hospital, New York, NY 10029, USA

## Abstract

*Objective.* To determine if there is an association between BMI and 3rd- or 4th-degree perineal lacerations in normal spontaneous and operative vaginal deliveries.* Study Design.* We performed a retrospective case control study using a large obstetric quality improvement database over a six-year period. Cases were identified as singleton gestations with third- and fourth-degree lacerations. Controls were obtained randomly from the database of patients without third- or fourth-degree lacerations in a 1 : 1 ratio. Univariate and multivariate logistic regression analyses were performed.* Results.* Of 32,607 deliveries, 22,011 (67.5%) charts with BMI documented were identified. Third- or fourth-degree lacerations occurred in 2.74% (*n* = 605) of patients. 37% (*n* = 223) were identified in operative vaginal deliveries. In the univariate analysis, obesity, older maternal age, non-Asian race, and birth weight <4000 g were all protective against 3rd- and 4th-degree lacerations. After controlling for age, race, mode of vaginal delivery, and birth weight, obesity remained significant.* Conclusion.* Being obese may protect against third- and fourth-degree lacerations independent of parity, race, birth weight, and mode of delivery.

## 1. Introduction

Obesity has reached epidemic proportions and affects most countries in the world. Obesity in the United States remains high at 36.5% of reproductive-age women of all ethnicities in 2012; 17% are class II or III obese, defined as BMI 35–39.9 kg/m^2^ and BMI of at least 40 kg/m^2^, respectively [1]. Given the variety of other risks obese women face during pregnancy (e.g., hypertensive disorders, gestational diabetes, complications of cesarean delivery, and fetal growth disturbances) [2–4], a more complete knowledge of what other risks they may encounter is invaluable. Obstetric lacerations are one area that has not been sufficiently studied.

Severe perineal lacerations are a serious complication of childbirth that can have both short and long term implications (e.g., incontinence, dyspareunia, acute and chronic pain, and even choice of cesarean delivery in subsequent pregnancies) [5]. Furthermore, quality control measurements of hospitals include third- and fourth-degree perineal lacerations, even though there may be patient characteristics that are unable to be controlled. Previously reported risk factors for third- and fourth-degree lacerations include Asian race, nulliparity, midline episiotomy, older maternal age, larger newborn birth weight, and assisted vaginal deliveries [6, 7]. Currently, there is limited literature regarding an association between lacerations and maternal obesity. It has been examined specifically in only one other study that is limited by its homogenous population of women [8]. Therefore, our objective was to observe if there is an association between BMI and third- and fourth-degree perineal lacerations in normal spontaneous and operative vaginal deliveries in a large database of an inner city and diverse population.

## 2. Materials and Methods

A retrospective study of a large administrative database was used to determine risk factors for 3rd- and 4th-degree lacerations after IRB approval was obtained. As part of quality improvement collaborative, the departments of obstetrics and gynecology at five academic medical centers, under the auspices of their common risk management advisors, FOJP Service Corporation, and their professional liability insurer, Hospitals Insurance Company (HIC), perform quarterly random chart audits at all affiliated institutions. This is an ongoing effort that began in January 2008. The charts of five women of each provider's deliveries per quarter are randomly selected by a computer as part of an effort to improve compliance with best obstetric practices as determined by a quality improvement committee of obstetric leaders from the participating hospitals. The audit involves 6 labor and delivery units spread across 3 New York City boroughs (Bronx, Manhattan, and Brooklyn). Nine individuals employed by HIC (all with clinical backgrounds and/or MPH degrees) perform the data abstraction.

Data is entered into a robust database designed to minimize data entry errors: most variables are preset drop-down menus or data buttons, and there is minimal free-text data entry. The sampling methodology is as follows: in each quarter, delivery logs from each hospital are obtained by HIC (the hospitals maintain logbooks listing all deliveries occurring on the labor and delivery service by date and time). Deliveries are entered into spreadsheets, and a random number generator is used to develop the sample population. The identified sample charts are then obtained from hospital medical records departments. HIC has electronic access for medical record reviews through secure electronic connections to the participating hospitals. While this is an ongoing auditing process, this analysis focused on the period from January 2008 to July 2013.

The data is maintained at HIC in a network password-protected database. Data collected in the analysis included age, gravidity, parity, race, gestational age, BMI on admission, diabetes, chronic hypertension, smoking history, oxytocin use, type of delivery, type of laceration, estimated fetal weight, birth weight, shoulder dystocia, and length of the second stage of labor (defined as time of being fully dilated to delivery time). Data on the use of episiotomy was not collected as part of this quality improvement initiative and therefore was not available to be included in the analysis. It is an uncommon practice in the participating institutions. The database was only set up to capture the occurrences of a 3rd- or 4th-degree laceration; it did not capture which type of laceration it was. For the purposes of the study, inclusion criteria included all vaginal deliveries of live, term, singleton, and vertex births; cesareans, multiple gestations, and records with an admission BMI missing were excluded. Cases were found through database query for third- and fourth-degree lacerations and controls were randomly selected from the database by computer randomization in a 1 : 1 ratio.

The primary outcome was third- or fourth-degree perineal lacerations. Student's *t*-test was used to compare continuous variables as appropriate, and *χ* ^2^ analysis was used to compare categorical variables. Multivariate logistic regression was then used to control for possible confounders with variables entered into the model that, in the univariate analysis, were significantly associated with the primary outcome variable with a *p* value > 0.01. Obesity was defined as a dichotomous variable defined as BMI ≥ 30 kg/m^2^ using World Health Organization (WHO) criteria. Individual classes of obesity were also assessed but were not powered for this study.

## 3. Results

Records of 32,601 deliveries were reviewed and 21,825 (66.9%) charts of singleton patients who delivered vaginally with BMI documented were identified. The overall rate of vaginal delivery was 67% (14,623) with an overall 7.4% (1,615) rate of operative vaginal delivery. Third- or fourth-degree lacerations were found in 605 patients (2.78%); 225 (37%) were identified in operative vaginal deliveries and 380 (63%) in nonoperative deliveries. The baseline demographics and clinical characteristics of the 1210 total women in the cases and the controls are presented in Table 1. The number of women in specific BMI categories is presented in Table 2.

Univariate analysis revealed that obesity, race, operative vaginal delivery, parity, birth weight, oxytocin use, shoulder dystocia, and length of second stage were all significantly associated with severe perineal lacerations. Specifically, third- and fourth-degree lacerations were more frequently found in women who were Asian, married, and nulliparous as well as among those in which oxytocin was administered, whose newborn's birth weight was more than 4000 g, and whose delivery was complicated by a shoulder dystocia (see Table 1). Women with operative vaginal deliveries were more likely to have a vaginal laceration (*p* < 0.0001). Severe lacerations were found less commonly in obese women (*p* = 0.0032). There were no significant differences between groups in mean age, smoking status, presence of chronic hypertension or diabetes, and the estimated fetal weight.

Multivariate analysis was then performed; the results are presented in Table 2. This analysis controlled for obesity, race, operative vaginal delivery, parity, birth weight, oxytocin use, shoulder dystocia, and length of second stage. Controlling for estimated fetal weight instead of birth weight did not yield a significant difference in the findings below. Obesity was still found to be inversely associated with third- and fourth-degree lacerations (OR 0.75, 95% CI 0.58–0.98). Birth weight <4000 g was also protective (OR 0.46, 95% CI 0.29–0.72) and the group without lacerations had a shorter mean second stage (OR 0.42, 95% CI 0.27–0.66). The following were still significantly associated with a greater incidence of severe perineal lacerations: Asian race (OR 1.65 (1.16–2.34)); nulliparity (OR 3.93 (2.98–5.19)); and operative vaginal delivery (OR 1.56 (1.20–2.04)). Oxytocin use was no longer significantly associated with these lacerations.

We also repeated our analysis defining obesity by WHO classifications. Individually, each class was not statistically associated with a lower risk of severe perineal laceration (Table 3), but the trend of increasing BMI class being associated with a lower risk of laceration was significant in our logistic regression (*p* = 0.037).

## 4. Discussion

We observed, in this large case control study, that maternal obesity was associated with a significantly lower incidence of severe perineal lacerations. Obesity is known as a risk factor for many negative pregnancy outcomes including but not limited to an increase in cesarean delivery rate, stillbirth, diabetes, and preeclampsia [2–4] but may be protective against severe perineal lacerations. Perineal lacerations specifically in obese women have been poorly studied; only one other study has addressed this issue. Lindholm and Altman found a correlation with a decreased risk of anal sphincter lacerations in obese women in a large database of Swedish women [8].

Other studies not specifically studying obese women have found mixed results; Hamilton et al. found a small decreased incidence of severe lacerations associated with BMI. This study used classification and regression trees to provide a specific risk that could be applied to everyday clinical practice. The authors found that a BMI defined as ≥26.7 kg/m^2^ was associated with a slightly decreased risk of third- and fourth-degree lacerations (OR 0.97) [9]. Hirayama et al. performed a cross-sectional study among 24 different countries and found no association between BMI and third- and fourth-degree lacerations [10]. Interestingly, Landy et al. found that increasing maternal BMI was protective in nulliparous and not in multiparous women [7]. They performed a large retrospective study from the Consortium on Safe Labor among 12 institutions looking at characteristics associated with third-degree, fourth-degree, and cervical lacerations [7].

Our study's findings are similar to those reported by Lindholm and Altman who also specifically studied the association between BMI and severe lacerations [8]. We have found a decrease in the risk for obstetric third- and fourth-degree lacerations that persisted in a multivariate analysis. An additional strength of our study is that it consisted of a diverse population potentially making it more generalizable.

Our speculations as to why obesity may be protective include the following: a different composition of the tissue in the perineum that may allow for more stretching [10], less force and frequency of uterine contractions contributing to a decrease in excessive contractions and subsequent pelvic floor injury [11], and possibly the maternal position of birth (e.g., squatting position use is less frequent [7, 12]). Damage to the perineum becomes increasingly important when considering that damage to the anal sphincter is a main contributing factor to anal incontinence as well as urgency symptoms [5]. Even after repair, many women have symptoms of fecal and flatal incontinence profoundly affecting health and self-image [5, 10, 13]. If obese women are having higher rates of anal and urinary continence issues, studies such as ours may inform the emphasis on commonly accepted etiologies; it may not be as likely to come from birth trauma as it is instead to come from the increased intra-abdominal pressure, dietary habits, and other comorbidities.

Our study's strengths include a large and diverse population of obese women, who were identified from major urban academic centers across New York City, making our results more generalizable to the contemporary North American population. We also have limitations. Our database is limited by the lack of information about episiotomy use. Although we know that episiotomies have become uncommon now, this study would be better served if this data was included. Future studies should include this information. In any observational study, there may be a risk for unmeasured confounding with risk factors that are unknown and could influence the relationship between risk factors and severe lacerations. There is also a risk of bias introduced by the women that did not have a BMI documented and therefore were not included in our study, but we believe that this risk is low since a failure to record BMI in the chart was most likely due to a random documentation error as opposed to some sort of systematic omission that might have some relationship with risk for perineal laceration. We recognize that the number of women having a third- or fourth-degree laceration was less than expected and at a lower rate than that noted in most of the other studies [4–16]. The rate of operative vaginal delivery was lower than most other studies as well. We also did not have information about the length of the perineal body or previous sphincter tear. There may be bias introduced in the identification of the degree of laceration by the providers; obesity might make visual recognition of lacerations, particularly third-degree tears, more challenging. Increased late sphincter retraction (a marker for inadequate repair) specific to obesity status has not been reported. A delivering provider might also underreport third- and fourth-degree defects if aware that these are safety indicators with potential professional and economic consequences for providers and hospitals [17]; this would be expected to decrease the incidence in the study population, although not preferentially in the obese.

It is important to remember that obstetrical trauma is part of the quality indicators suggested by the Agency for Healthcare Research and Quality (AHRQ). When evaluating hospitals for these indicators, the variability between hospitals may not be related to the hospital but rather to the patient population [17]. Our findings, consistent with the only other study specifically addressing obesity and maternal obstetric trauma, support the assertion that the use of severe perineal lacerations as a quality outcome measure should be adjusted appropriately for patient characteristics.

In summary, our large and urban case control study provides evidence that obesity may be protective against the risk of third- and fourth-degree lacerations. Larger studies of obese women that include the use of episiotomy data would be helpful. Considering all evidence, including a decreased laceration risk in obese patients, allows us to question the value of third- and fourth-degree lacerations as a quality indicator. Deeper understanding of the nonmodifiable population characteristics in quality measures will only help to develop better and more valid obstetric quality indicators in the future.

## Figures and Tables

**Table 1:** Demographics and clinical characteristics of the study sample (*n* = 1210).

Demographic or clinical characteristic	No laceration *n* = 605	Laceration *n* = 605	*p*
Maternal age (y)	28.6 ± 6.1	29.0 ± 6.0	NS
Marital status			0.02
Married	376 (62.2)	387 (64.0)	
Single/never married	215 (35.5)	188 (31.0)	
Other	14 (2.3)	30 (5.0)	
Race/ethnicity			<0.0001
Caucasian	241 (39.8)	228 (37.7)	
African American	85 (14.1)	66 (10.9)	
Hispanic	151 (25.0)	101 (16.7)	
Asian	87 (14.4)	145 (24.0)	
Other	41 (6.7)	65 (10.7)	
Smoking			NS
No	551 (91.1)	572 (94.6)	
Prior	20 (3.3)	11 (1.8)	
Current	17 (2.8)	11 (1.8)	
Unknown	17 (2.8)	11 (1.8)	
Obese (BMI ≥ 30 kg/m^2^)	261 (43.0)	211 (34.9)	0.0032
Parity			<0.0001
Multiparity	316 (52.2)	140 (23.2)	
Nulliparity	289 (47.8)	465 (76.8)	
Oxytocin use	293 (48.4)	373 (61.7)	<0.0001
Estimated fetal weight ≥ 4000 g	13 (2.5)	24 (4.4)	NS
Birthweight ≥ 4000 g	32 (5.3)	77 (12.8)	<0.0001
Chronic hypertension	40 (6.6)	56 (9.3)	NS
Diabetes (all)	45 (7.5)	56 (9.3)	NS
Operative VD^*∗*^	1394 (6.6)	225 (37.2)	<0.0001
Shoulder dystocia	32 (5.3)	82 (13.6)	<0.0001
Second stage (hr)	1.3 ± 2.3	1.9 ± 2.1	<0.0001

BMI: body mass index.

VD: vaginal delivery.

NS = not significant.

Data are mean ± standard deviation or *n* (%).

^*∗*^OVD calculated from overall acceptable group (*n* = 21,825).

**Table 2:** Multivariate logistic regression for predicting risk of laceration.

Variable	aOR (95% CI)	*p* value
Obesity (BMI ≥ 30 kg/m^2^)	0.75 (0.58–0.98)	0.037
Asian race	1.65 (1.16–2.34)	0.002
African American race	0.67 (0.45–0.99)	0.001
Operative VD	1.56 (1.20–2.04)	0.001
Nulliparity	3.93 (2.98–5.19)	<0.001
Birthweight < 4 kg	0.46 (0.29–0.72)	0.0007
No prolonged second stage	0.42 (0.27–0.66)	0.0002

BMI: body mass index.

VD: vaginal delivery.

aOR: adjusted odds ratio.

CI: confidence interval.

**Table 3:** Multivariate analysis^*∗*^ of BMI distribution for prediction of risk of laceration.

BMI class	No laceration	Laceration	aOR (95% CI)
Overweight (25–29.9 kg/m^2^)	262 (46)	308 (54)	0.99 (0.68–1.44)
Obese (30+ kg/m^2^)			
Class I (30–34.9 kg/m^2^)	166 (52)	155 (48)	0.84 (0.55–1.28)
Class II (35–39.9 kg/m^2^)	61 (60)	40 (40)	0.58 (0.33–1.02)
Class III (40+ kg/m^2^)	33 (67)	16 (33)	0.52 (0.25–1.1)

Data are presented as *n* (%)

^*∗*^Variables that were controlled for were race, OVD, birth weight categories, Pitocin use, and shoulder dystocia.
